# Spatio-temporal variations of licensed doctor distribution in China: measuring and mapping disparities

**DOI:** 10.1186/s12913-020-4992-2

**Published:** 2020-03-02

**Authors:** Bin Zhu, Chih-Wei Hsieh, Ying Mao

**Affiliations:** 1 School of Public Policy and Administration, Xi’an Jiaotong University, 28 Xianning West Road, Beilin District, Xi’an, 710049 China; 2 Department of Public Policy, City University of Hong Kong, Tat Chee Avenue, Kowloon, Hong Kong, SAR China

**Keywords:** Licensed doctor distribution, Spatio-temporal variations, Temporal trends, Spatio-temporal clusters, Moran’s I, Space-time scan, China

## Abstract

**Background:**

The licensed doctor misdistribution is one of the major challenges faced by China. However, this subject remains underexplored as spatial distribution characteristics (such as spatial clustering patterns) have not been fully mapped out by existing studies. To fill the void, this study aims to explore the spatio-temporal dynamics and spatial clustering patterns of different subtypes of licensed doctors (i.e., clinicians, traditional Chinese medicine doctors, dentists, public health doctors, general practitioners) in China.

**Methods:**

Data on the licensed doctor quantity and population during 2012–2016 was obtained from the National Health (and Family Planning) Yearbook. Functional boxplots were used to visualize and compare the temporal trends of densities of different subtypes of licensed doctors. This study adopted two complementary spatial statistics (space-time scan statistics and Moran’s I statistics) to explore the spatio-temporal dynamics and spatial clustering patterns of licensed doctor distribution in China. The former was used to explore the spatial variations in the temporal trends of licensed doctor density during 2012–2016, and the latter was adopted to explore the spatial changing patterns of licensed doctor distribution during the research period.

**Results:**

The results show that the densities of almost all subtypes of licensed doctors displayed upward trends during 2012–2016, though some provincial units were left behind. Besides, spatial distribution characteristics varied across different subtypes of licensed doctors, with the low-low cluster area of general practitioners being the largest.

**Conclusions:**

The misdistribution of licensed doctors is a global problem and China is no exception. In order to achieve a balanced distribution of licensed doctors, the government is suggested to introduce a series of measures, such as deliberative policy design and effective human resource management initiatives to educate, recruit, and retain licensed doctors and prevent a brain drain of licensed doctors from disadvantaged units.

## Background

In December 2015, 17 Sustainable Development Goals (SDGs) were adopted to provoke worldwide efforts to address global development issues for the coming 15 years [1]. In consonance with the Millennium Development Goals (MDGs), which were introduced in 2000 and have since contributed to improved human wellbeing around the globe, the SDGs also position health as a key feature of continuing human development but take the further step of establishing the goal of health improvement with 13 specific targets [2]. More effective human resource management in healthcare is one of the 13 targets, which is directed exclusively at developing countries and is now drawing the attention of the developing world including China.

As one of the major resource inputs, human resources are crucial for the proper functioning of any national healthcare system [3]. Many studies have shown a significant relationship between the density of healthcare workers and health outcomes. For instance, in an analysis of the global workforce, Chen and colleagues [4] reported that the increased density of healthcare workers in a population is associated with the improvement of population-based health and human survival. By examining 192 countries around the world, Speybroeck et al. [5] also found that the density of health manpower is negatively correlated with maternal mortality rates. World Health Organization (WHO) [6] has therefore used the density of skilled health professionals per 1000 population as an important indicator to assess the performance of a healthcare system.

With the concerted efforts of WHO, many countries have made several global, regional and national investments, most of which are centered on addressing concerns about the number of skilled healthcare workers [7]. In order to cope with the issue of health manpower shortage, China introduced a series of measures including the expansion campaign of medical and nursing schools in 1998 [8]. As a result of continuous efforts, significant changes in the quantity of health manpower have begun in China and the shortage of health manpower has gradually eased. According to the Health Statistical Yearbook [9] and Long-Term Medical Personnel Development Plan of China (2011–2020) [10], the number of licensed doctors per capita averaged the whole country (2.44 in 2017) has overfulfilled the 2020 target (2.10). However, the misdistribution of health manpower is becoming increasingly severe as most of the health workers are concentrated in eastern developed regions, which limits the health services accessibility in rural and remote China [11].

In existing studies, initial efforts have already been made to explain the misdistribution of health manpower. In some cases, the academic world explored this topic just by comparing the health manpower availability indicators in different regions. Tokuhata et al. [12] analyzed the distribution of physicians and other licensed health personnel in the state of Pennsylvania by comparing their densities in different counties. Ahmed et al. [13] compared the physician and nurse density in urban and rural Bangladesh by using national sample survey data. Gupta et al. [14] compared and mapped the geographical variations in health manpower distribution in Kenya, Mexico and Viet Nam and concluded the strong inequalities in health workforce distribution in these three countries. In other cases, the economic indicators were adopted to empirically measure the equity in health manpower distribution. For instance, Hazarika [15] investigated the inequalities in the distribution of doctors, dentists, nurses and midwives by calculating the Gini coefficient. Theodorakis et al. [16] measured the health inequalities in general practitioner distribution in Albania by plotting Lorenz curves and calculating Gini coefficients. Honarmand et al. [17] calculated the Gini coefficient and Robin Hood index to investigate the equity in general practitioners, midwives, pediatricians, and gynecologists distribution in Iran. Wiseman [18] adopted Lorenz Curve/Gini Coefficient and Theil Index to explore the inequality in health manpower distribution in Fiji for doctors, nurses, and all health manpower (doctors, nurses, dentists and health support staff).

Thus far, while there have been some empirical studies of the health manpower distribution in China, almost all existing studies tried to understand the status quo of the health manpower distribution through the lens of health equity. Anand and colleagues [8] calculated three measures (i.e., Gini coefficient, Theil-T, Theil-L) of inequity by using the density of health workers between different provinces and concluded that the misdistribution of health manpower occurs at various gradients, including urban-rural, regional and national levels. Chen et al. [19] also estimated the inequity in the distribution of community health manpower by considering population size and geographical area (i.e., Lorenz curve and Gini coefficient). In addition, Liu et al. [20] disaggregated data into three regions (west, central, and east) by geographical location and used Theil index to investigate the equity trend of health manpower distribution after the New Medical Reform launched in 2009.

Despite knowledge has accumulated, existing research is by and large based on the traditional economic methods which have significant defects. That is, the traditional economic methods only focus on the overall regional differences but ignored the space location information. As a result, detailed spatial distribution characteristics and spatio-temporal dynamics of health manpower distribution have not been explored in existing studies.

To fill the research void, this study proposed to explore the temporal trends, spatio-temporal dynamics and spatial cluster areas of different subtypes of licensed doctors, which are the most important component of health manpower in China. Compared to existing literature, this study applied spatial statistics so that the spatial distribution characteristics of licensed doctors can be fully analyzed. In this study, two complementary spatial statistics are introduced to display the detailed spatial distribution characteristics of health manpower, thus not only providing more evidence for understanding the health manpower distribution in China but also serving as a methodological reference for future studies of equity in the distribution of health manpower. In addition, this study further tracks the changes in spatial distribution characteristics of health manpower distribution over time. The panel dataset provides an opportunity to conduct a spatial analysis from two different analytical angles. The cross-sectional analysis explores the comparative features of health manpower distribution at the provincial level, while the longitudinal analysis reveals the spatial-temporal dynamics of health manpower distribution during the research period. We believe that the research finding can serve as a basis for addressing the misdistribution of licensed doctors in China.

## Methods

### Data resources

This study used the provincial-level year-end data in China during 2012–2016. Only the provincial administrative units in mainland China were included in this study. Hong Kong and Macau were excluded because of data unaccessibility. The data were obtained from China’s Health Statistical Yearbook, and China’s Health and Family Planning Statistical Yearbook. They were both published by the National Health Commission of China under its own data authority. The Chinese administrative divisions can be found in the Supplementary Fig. S1 All the original data adopted in this study can be found in the Supplementary Table S1 and Table S2.

### Measurement of the indicators

The total number of licensed doctors is an important indicator to evaluate licensed doctor availability in one region/country. However, as regions differ in population, the total number cannot be used for comparison across different regions or nations. To exclude the effect of population, the licensed doctor density is widely used as an indicator for regional comparison [21]. It is defined as the ratio of licensed doctor quantity to population size and conventionally calculated as the total licensed doctor per 1000 population (Formula 1). It is widely used in WHO reports, government statistical files, and academic research to evaluate the performance of a health system [22–25], and is also the only quantitative target indicator in the Long-Term Medical Personnel Development Plan of China (2011–2020) [10].

1 $$ Licensed\ doctor\ density=\frac{Health\ manpower\ \mathrm{n} umber}{\  Population}\times 1000 $$

According to the *China Health and Family Planning Statistical Yearbook 2017* [9], licensed doctors in China are doctors with the certificate of (assistant) medical practitioner who are engaged in medical practice. They comprise of clinicians, traditional Chinese medicine (TCM) doctors, dentists, public health doctors, while some licensed doctors own dual identity as they are also registered as general practitioners (GP) at the same time. Therefore in total there are 5 indicators of licensed doctors included in this study (Table 1).

**Table 1:** Indicators included in this study

Indicators	Calculation
Clinician density	$\frac{clinician\ number}{\ population}\ast 1000$
Traditional Chinese medicine (TCM) doctor density	$\frac{TCM\ doctor\ number}{\ population}\ast 1000$
Dentist density	$\frac{dentist\ number}{\ population}\ast 1000$
Public health doctor density	$\frac{public\ health\ doctor\ number}{\ population}\ast 1000$
General practitioner density	$\frac{general\ practitioner\ number}{\ population}\ast 1000$

### Analytical tools

This study adopted two complementary spatial statistics (space-time scan statistics and Moran’s I statistics) to explore the spatio-temporal dynamics and spatial clustering patterns of licensed doctor distribution in China. The former was used to explore the spatial variations in the temporal trends of licensed doctor density during 2012–2016, and the latter was adopted to explore the spatial changing patterns of licensed doctor distribution during 2012–2016.

#### Space-time scan statistics

Kulldorff’s space-time scan statistic explores the spatial variations in temporal trends of licensed doctor densities. More specifically, it identifies those units in which the rates of change of licensed doctor density are significantly higher or lower than the others. Kulldorff’s space-time scan statistic is defined by a circular window with a geographic base [26]. The first step of space-time scan analysis is to impose the window on the map that is then moved in space and time. The window visits each possible geographical position with each possible window size. The null hypothesis is that the trends of geographical units in different windows are the same, while the alternative hypothesis is that the trends are different. On the basis of the hypotheses, the difference in the rates of change inside and outside the windows is evaluated by the Log Likelihood Ratio (LLR, Formula 3.2) [27], which is lower the more likely it is that the trend difference is due to chance.

2 $$ \mathrm{LLR}=\log \left\{{\left(C/n\right)}^c{\left[\left(C-c\right)/\left(C-n\right)\right]}^{\left(C-c\right)}\right\} $$

*C: Total change rate of licensed doctor density.*

*c: Observed change rate of licensed doctor density inside the space-time scan window.*

*n: Expected change rate of licensed doctor density inside the space-time scan window.*

**Fig. 1:**
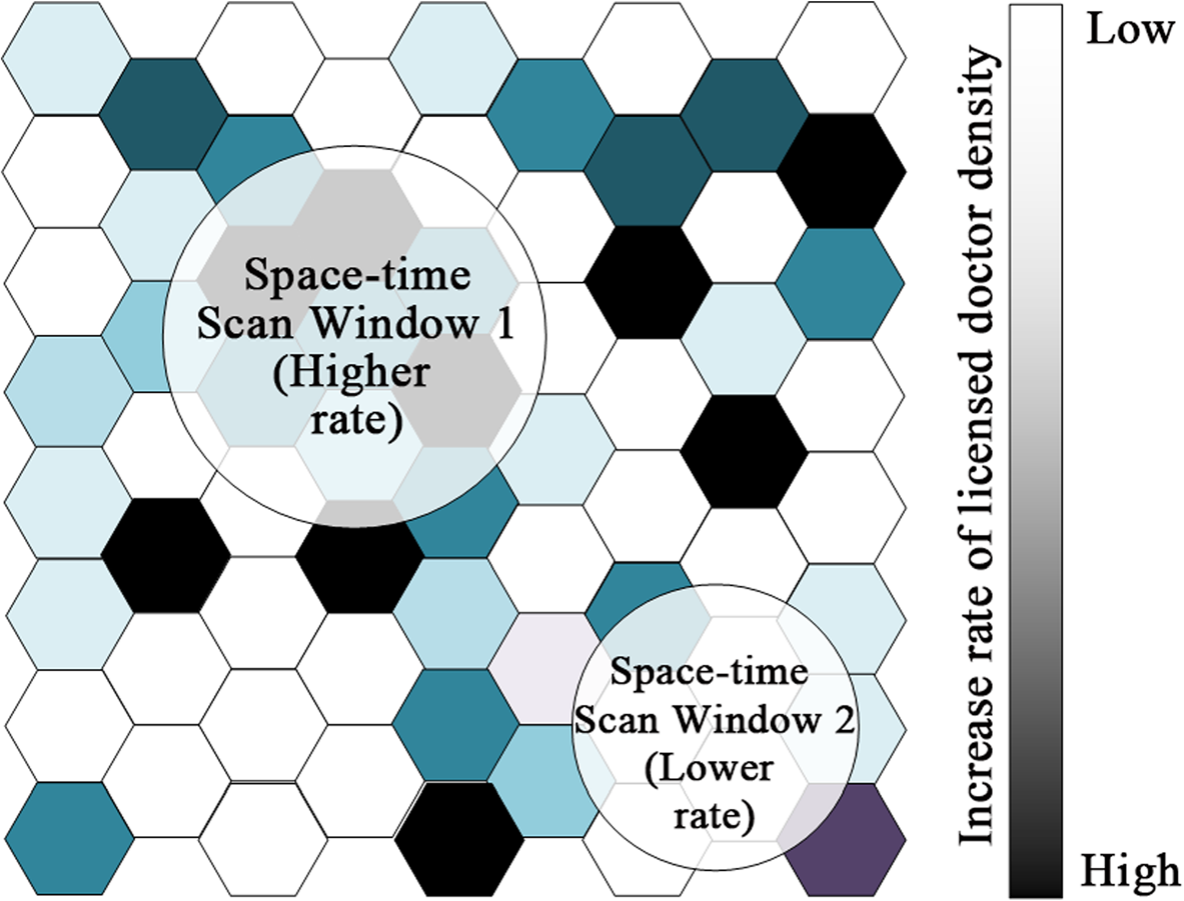
Two types of spatial clusters detected by Kulldorff’s space-time scan statistics

Based on the values of the LLR, the space-time scan statistic can identify both the higher and lower-rate clusters (Fig. 1, the rate of change of the licensed doctor density within the geographical units inside the window is higher or lower, respectively, than that of units outside of the window). For either higher-rate or lower-rate clusters, the window with the largest LLR is referred to as the most likely cluster for which the trend inside the window is most unlikely to be the same as the units outside the window, while others (if any) are known as secondary clusters. For this analysis, a Poisson-based model was used and Monte Carlo randomization (999 permutations) was employed to compute the significance of the Kulldorff’s space-time scan statistics.

#### Local Moran’s I

Spatial autocorrelation, which means the correlation among values of a single variable resulting from the proximity of those values in geographical space [28], particularly targets the spatial disparities resulting from the geographical location. Moran’s I is one of the most common spatial autocorrelation indicators [29–31], which has the unique advantage of describing and visualizing geographical distributions and identifying units or subset of units that are unusual. There are two forms of Moran’s I—global Moran’s I and local Moran’s I.

Global Moran’s I is a measure describing the overall spatial distribution characteristic of the whole area [31, 32]. Therefore, only one value is calculated for the entire sample. Local Moran’s I, which is also referred to as the local indicator of spatial association (LISA), is an indicator for a particular area. It can be regarded as the result of the decomposition of Global Moran’s I [29]. It is defined as

3 $$ {\mathrm{Local}\ \mathrm{Moran}}^{\prime}\mathrm{I}=\frac{\left({y}_i-\overline{y}\right)}{m_0}{\sum}_j{W}_{ij}\left({y}_j-\overline{y}\right)\kern1.25em {m}_0={\sum}_j{\left({y}_i-\overline{y}\right)}^2/n $$

*n: the number of geographical units (31 provincial units in this study).*

*y*_*i*_ *: the density of one certain type of licensed doctors in geographical unit i.*

*y*_*j*_ *: the density of one certain type of licensed doctors in geographical unit j.*

$\overline{y}$ *: the average density of one certain type of licensed doctors in all the geographical units.*

*W*_*ij*_:*spatial-weighted n × n matrix which represents neighboring relations. W*_*ij*_ *= 1 if unit i is adjacent with unit j, and W*_*ij*_ *= 0 otherwise.*

**Fig. 2:**
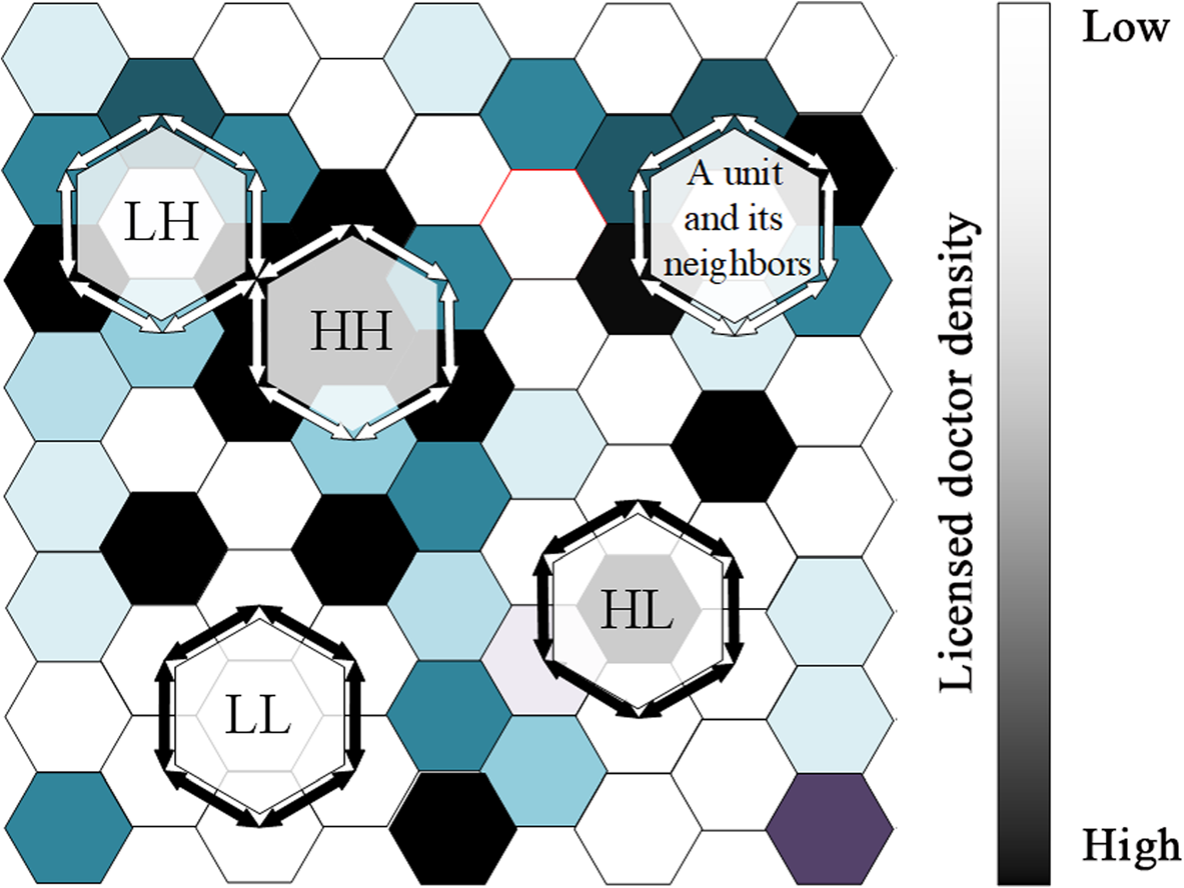
Four types of spatial clusters (HH, HL, LH, LL) detected by local Moran’s I

The operation of summing over unit *j* is limited to the neighbors of unit *i* [33]. A positive value of local Moran’s I indicates a resemblance between one geographical unit and its adjacent areas, whereas a negative value implies a dissimilarity between units [33]. Normally it is used to identify outliers and leverage points, i.e., units with statistical significance. Based on its value and significance, the local Moran’s I can detect four types of clusters (Fig. 2), reflecting the high-low (HL, geographical units with high values surrounded by geographical units with low values), high-high (HH, geographical units with high values surrounded by geographical units with high value), low-low (LL, geographical units with low values surrounded by geographical units with low values) and low-high (LH, geographical units with low values surrounded by geographical units with high values) clustering patterns. In this context, the identification of spatial clusters indicate that the licensed doctor distribution characteristics has gone beyond the provincial level and exhibits a trend of regionalization. Take the LH and LL clusters as examples, low-high cluster type means that the central unit is at a disadvantage when compared to its neighbors, low-high cluster type means the central unit and its neighbors are confronting the manpower shortage at the same time. As the spatial cluster features of licensed doctors vary across space, the outliers and leverage points that display fairly unique characteristics have relatively abundant or inadequate licensed doctors, and thus should be precisely the places on which to focus to promote an equitable distribution.

To illustrate statistically significant spatial autocorrelations (also known as spatial clusters) on China’s geographical map, a graph which displays those units whose Local Moran’s I passes the significance level (i.e., α = 0.05) will be presented in a later section. As marked areas highlight positive or negative spatial autocorrelations, this graph is known as the univariate LISA cluster map. In order to visualize the dynamics of licensed doctor distribution more effectively and concisely, three equidistant time points 2012, 2014, 2016 are selected to display the univariate LISA cluster map. Besides, the hierarchical maps of the corresponding indicator are also be displayed alongside the cluster map for reference. In the hierarchical maps, the density of one certain type of licensed doctors is classified into five classes, which are displayed in different colors. The class breakdown in the hierarchical maps is based on the maximum and minimum of licensed doctor density during 2012, 2014 and 2016. The differences between these two values are divided evenly into five categories. For instance, if the maximum and minimum are 20 and 0 respectively, then the five classes are 0–4, 4–8, 8–12, 12–16, and 16–20. After that, the data were loaded into China’s administrative map with provincial boundaries indicated.

### Software

The local Moran’s I was computed using GeoDa 1.12.1 [34]. The space-time scan analysis was conducted with SatScan 9.5 (Kulldorff and Information Management Services, Inc., Boston, MA, USA). Hierarchical maps and univariate LISA maps were developed with ArcGIS 10.0. The descriptive functional boxplots were made with Microsoft Excel 2016 (Microsoft Corporation, Redmond, WA, USA).

## Results

### Descriptive statistics

**Fig. 3:**
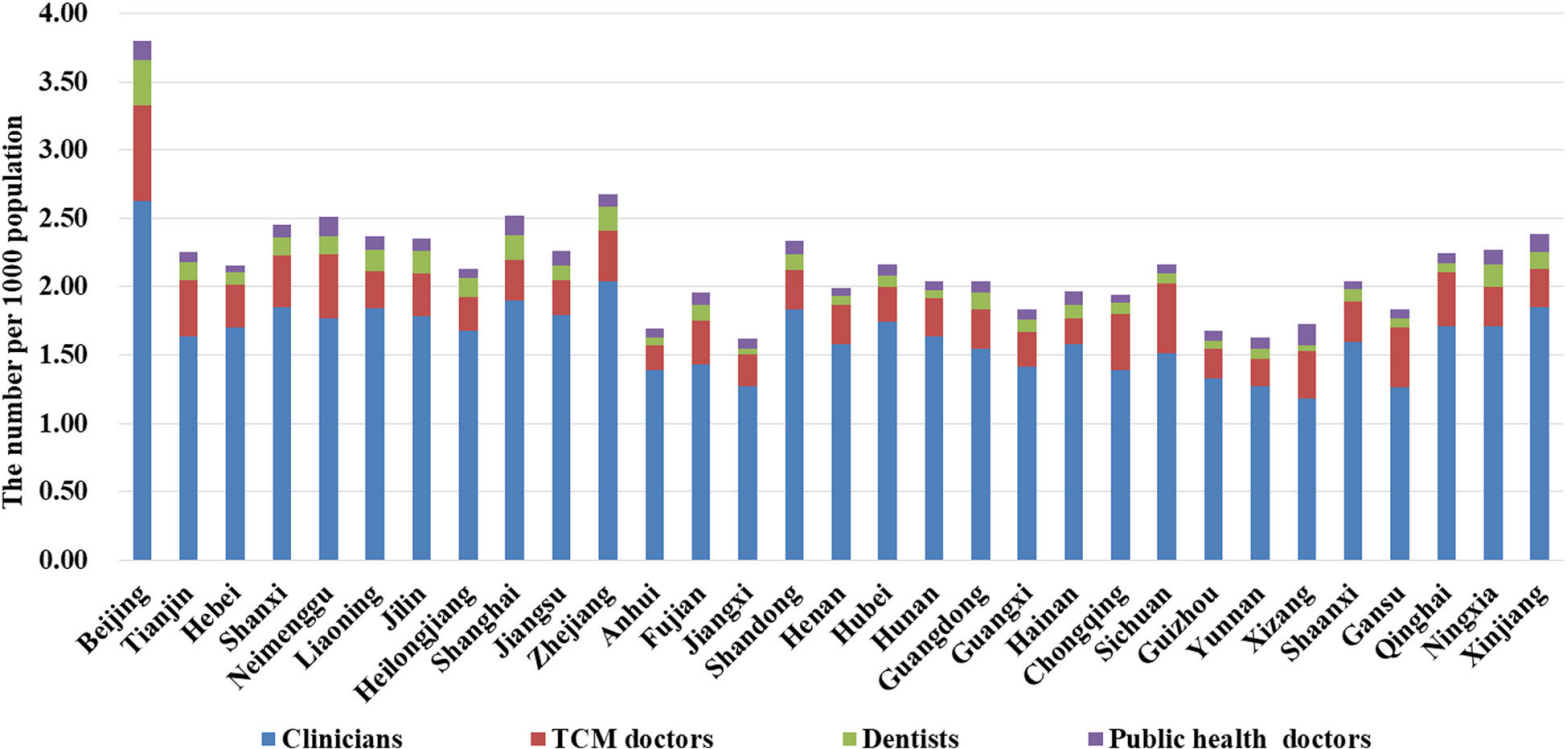
Average densities of different subtypes of licensed doctors during 2012–2016

Figure 3 shows the average densities of all subtypes of licensed doctors in each provincial unit in China and the original data are attached below the figure. As for its four components, clinicians accounted for the largest proportion of licensed doctors in all the provincial units, while the density of public health doctors was usually smaller when compared to dentists and TCM doctors in a provincial unit. Beijing owned the highest density of all subtypes of licensed doctors, while the lowest average densities of clinicians, TCM doctors, dentists, and public health doctors were located in Xizang, Anhui, Xizang, Hebei, respectively. In general, only a small proportion (usually less than 10%) of licensed doctors registered as general practitioners at the same time.

### Temporal trends

**Fig. 4:**
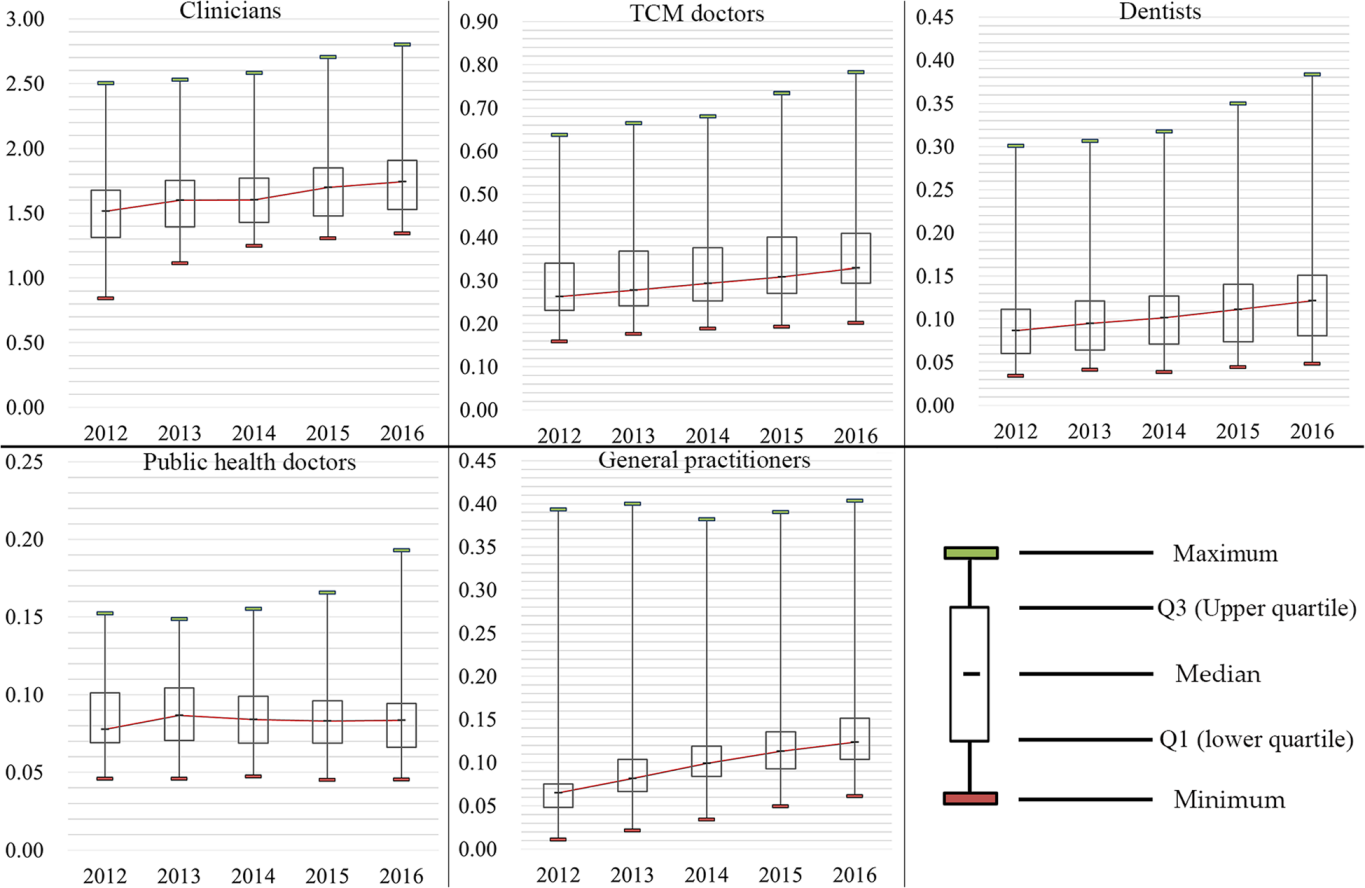
Boxplots of the density of licensed doctors during 2012–2016

Figure 4 displays the temporal trends of the densities of different subtypes of licensed doctors during 2012–2016. Generally speaking, China experienced a rapid increase in the density of almost all subtypes of licensed doctors, which was evidenced by the upswept trend lines. The density of public health doctors was the only subtype which experienced decreases in the median. Intriguingly, the general practitioner density displayed a positively skewed distribution (For a unimodal distribution, negative skew commonly indicates that the tail is on the left side of the distribution, and positive skew indicates that the tail is on the right.), meaning that some units have much higher densities than the rest.

### Spatial variations in temporal trends

**Fig. 5:**
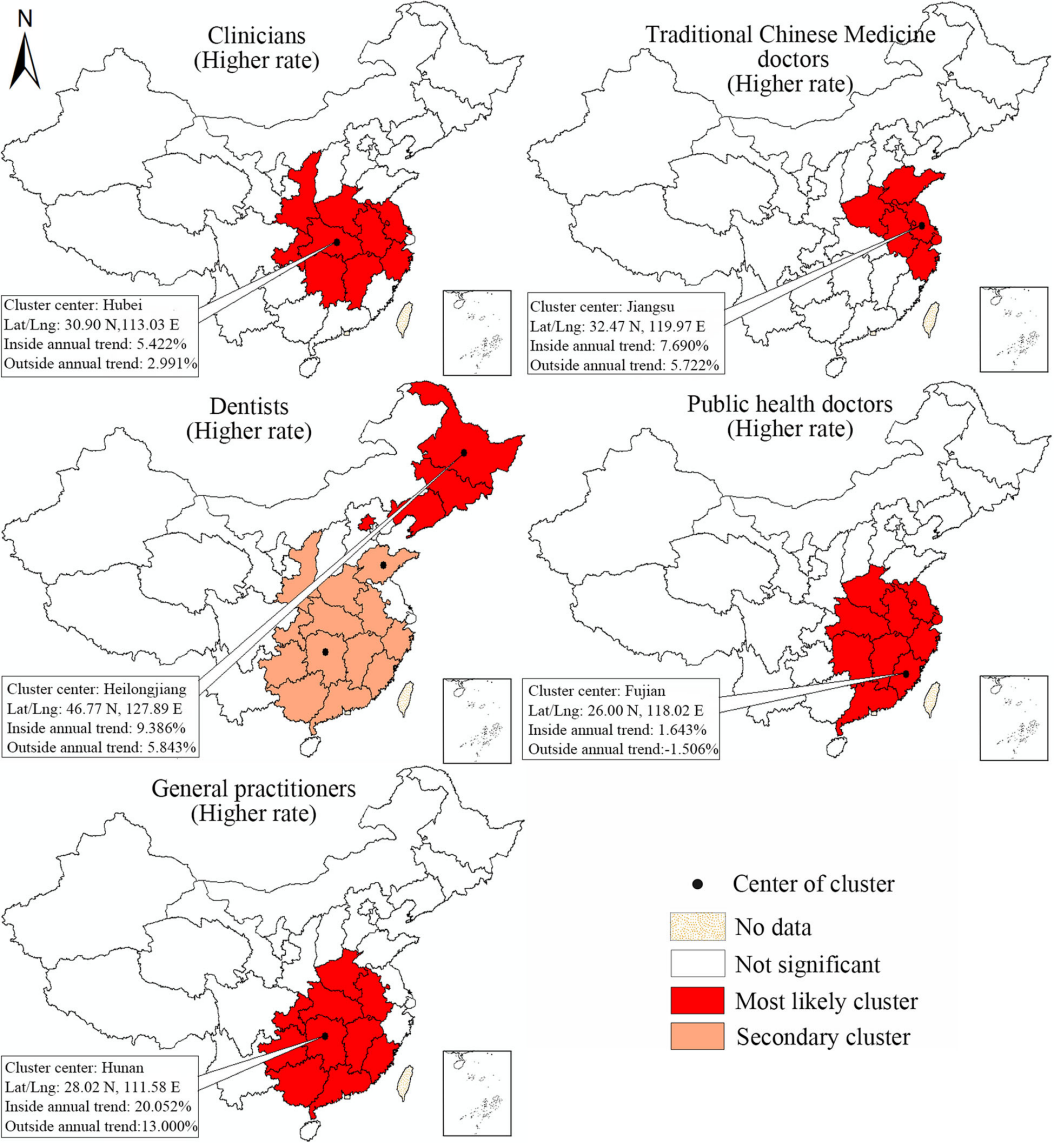
Space-time clusters of higher change rate of densities by subtype of licensed doctors. (Note: Detailed information of the most likely clusters is noted on the maps)

Figure 5 visualizes the space-time clusters of higher change rate of densities broken down by subtype of licensed doctors. In short, the maps differ greatly in the units that included in the clusters. The most likely cluster of higher change rate of clinicians was located in 9 provincial units in the middle part of China, with Hubei being the cluster center. The mean annual change in rate for these 9 provincial units reached 5.422%, which was almost twice of that in other units (2.991% annually). An accelerated increase of TCM doctor density was observed in Jiangsu (center), Shandong, Henan, Shanghai, Anhui, Zhejiang, with an average annual growth rate reaching 7.690%.

As for the dentists, Heilongjiang was detected as the center of the most likely cluster, which also included Jilin, Liaoning and Beijing. A 9.386% average annual increase rate was observed in these units. In addition, almost all the units in middle and eastern China displayed a faster growth of dentist density than western China, which was evidenced by two secondary clusters that included 13 units. In terms of the public health doctors, its density in 10 provincial units (center: Fujian) in southeast China increased 1.643% annually during 2012–2014, while a decreasing trend (− 1.506% annually) was observable outside the most likely cluster. Regarding the general practitioners, a significantly higher increase of its density was observed in 10 provincial units in middle and southern China (20.052% versus 13.000% annually).

**Fig. 6:**
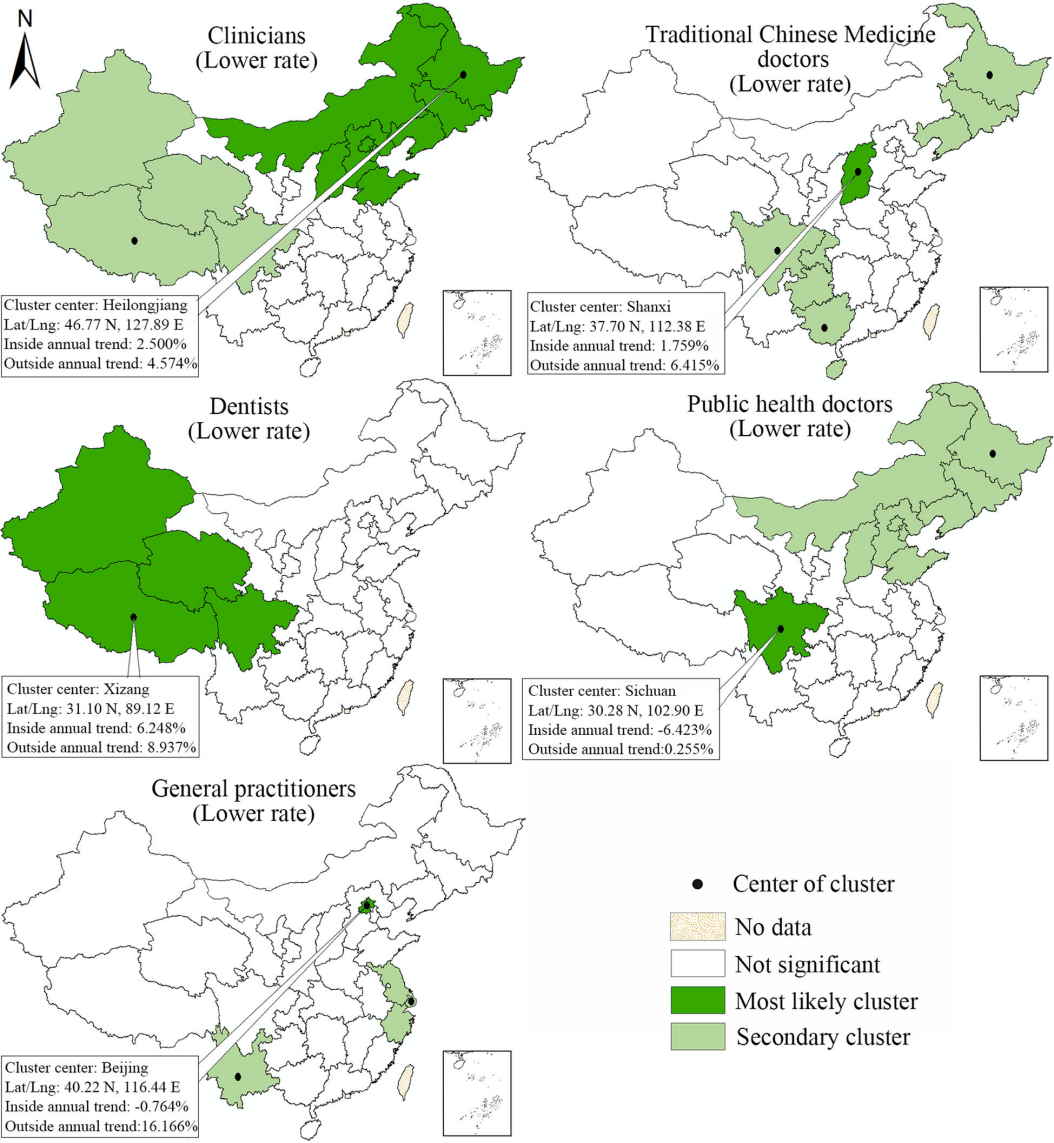
Space-time clusters of lower change rate of densities by subtype of licensed doctors. (Note: Detailed information of the most likely clusters is noted on the maps)

Figure 6 visualizes the space-time clusters of lower change rate of densities broken down by subtype of licensed doctors. Two clusters which displayed significant lower increases were identified for clinician density. The most likely cluster comprised 9 units in northwest China (center: Heilongjiang), while the secondary cluster was situated in 4 remote western units (Xinjiang, Xizang, Qinghai, Sichuan), with Xizang being the cluster center. Similarly, a slower increase in dentist density was also detected in these four provincial units, the 6.248% annual increase rate among which was significantly slower than the observed in other units (8.937% annually). As for the TCM doctors, public health doctors and general practitioner, the most likely clusters of lower change rate were only detected in one provincial unit, being Shanxi, Sichuan and Beijing, respectively. It was noteworthy that the public health doctor density in Sichuan even decreased 6.423% annually, which was in sharp contrast to the 0.255% annual increase among other 30 provincial units. More information about all the space-time clusters of higher and lower increase rates of health technician density can be found in Table 2.

**Table 2:** Space-time clusters of higher and lower rates of change of densities by subtype of licensed doctors during 2012–2016

Manpower	Cluster type	Units included	Coordinates of cluster center	Radius of cluster (Km)	Average density	Inside time trend (Annual)	Outside time trend (Annual)	LLR	p
Clinician*	Higher	Hubei (center), Henan, Hunan, Anhui, Jiangxi, Chongqing, Shaanxi, Jiangsu, Zhejiang	30.90 N, 113.03 E	709.15	1.625	5.422%	2.991%	1435.02	0.001
Clinicians*	Lower	Heilongjiang (center), Jilin, Liaoning, Beijing, Tianjin, Hebei, Shandong, Neimenggu, Shanxi	46.77 N, 127.89 E	1620.87	1.823	2.500%	4.574%	956.88	0.001
Clinicians	Lower	Xizang (center), Qinghai, Xinjiang, Sichuan	31.10 N, 89.12 E	1319.29	1.581	1.931%	4.099%	366.38	0.001
TCM doctor*	Higher	Jiangsu (center), Shanghai, Anhui, Zhejiang, Shandong, Henan	32.47 N, 119.97 E	611.70	0.279	7.690%	5.722%	142.04	0.001
TCM doctor*	Lower	Shanxi (center)	37.70 N, 112.38 E	0	0.383	1.759%	6.415%	135.03	0.001
TCM doctor	Lower	Heilongjiang (center), Jilin, Liaoning	46.77 N, 127.89 E	683.82	0.276	4.004%	6.426%	74.35	0.001
TCM doctor	Lower	Guangxi (center), Hainan, Guizhou	23.02 N, 108.41 E	444.67	0.233	8.957%	6.118%	70.25	0.001
TCM doctor	Lower	Sichuan (center), Chongqing	30.28 N, 102.90 E	471.25	0.485	5.560%	6.356%	13.27	0.001
Dentist*	Higher	Heilongjiang (center), Jilin, Liaoning, Beijing	46.77 N, 127.89 E	1172.90	0.180	9.386%	5.843%	105.95	0.001
Dentist	Higher	Hunan (center), Hubei, Jiangxi, Chongqing, Guizhou, Guangdong, Guangxi, Henan, Fujian, Anhui, Shaanxi, Zhejiang	28.02 N, 111.58 E	840.52	0.088	10.061%	7.806%	73.27	0.001
Dentist	Higher	Shandong (center)	36.18 N, 118.43 E	0	0.119	12.502%	8.412%	72.33	0.001
Dentist*	Lower	Xizang (center), Qinghai, Xinjiang, Sichuan	31.10 N, 89.12 E	1319.29	0.086	6.248%	8.937%	28.32	0.001
Public health doctor*	Higher	Fujian (center), Jiangxi, Zhejiang, Guangdong, Anhui, Hunan, Shanghai, Hubei, Jiangsu, Henan	26.00 N, 118.02 E	965.93	0.081	1.643%	−1.506%	137.03	0.001
Public health doctor*	Lower	Sichuan (center)	30.28 N, 102.90 E	0	0.063	−6.423%	0.255%	116.05	0.001
Public health doctor	Lower	Heilongjiang (center), Jilin, Liaoning, Beijing, Tianjin, Hebei, Shandong, Neimenggu, Shanxi	46.77 N, 127.89 E	1620.87	0.088	−2.146%	0.845%	105.81	0.001
Gereral practitioner*	Higher	Hunan (center), Hubei, Jiangxi, Chongqing, Guizhou, Guangdong, Guangxi, Henan, Fujian, Anhui	28.02 N, 111.58 E	698.79	0.094	20.052%	13.000%	649.50	0.001
Gereral practitioner*	Lower	Beijing (center)	40.22 N, 116.44 E	0	0.388	−0.764%	16.166%	968.41	0.001
Gereral practitioner	Lower	Shanghai (center), Jiangsu, Zhejiang	31.21 N, 121.68 E	279.11	0.283	12.777%	16.277%	149.60	0.001
Gereral practitioner	Lower	Yunnan (center)	24.14 N, 101.30 E	0	0.087	7.162%	15.478%	110.84	0.001

Note:Most likely clusters are noted with *

### Spatial changing patterns

**Fig. 7:**
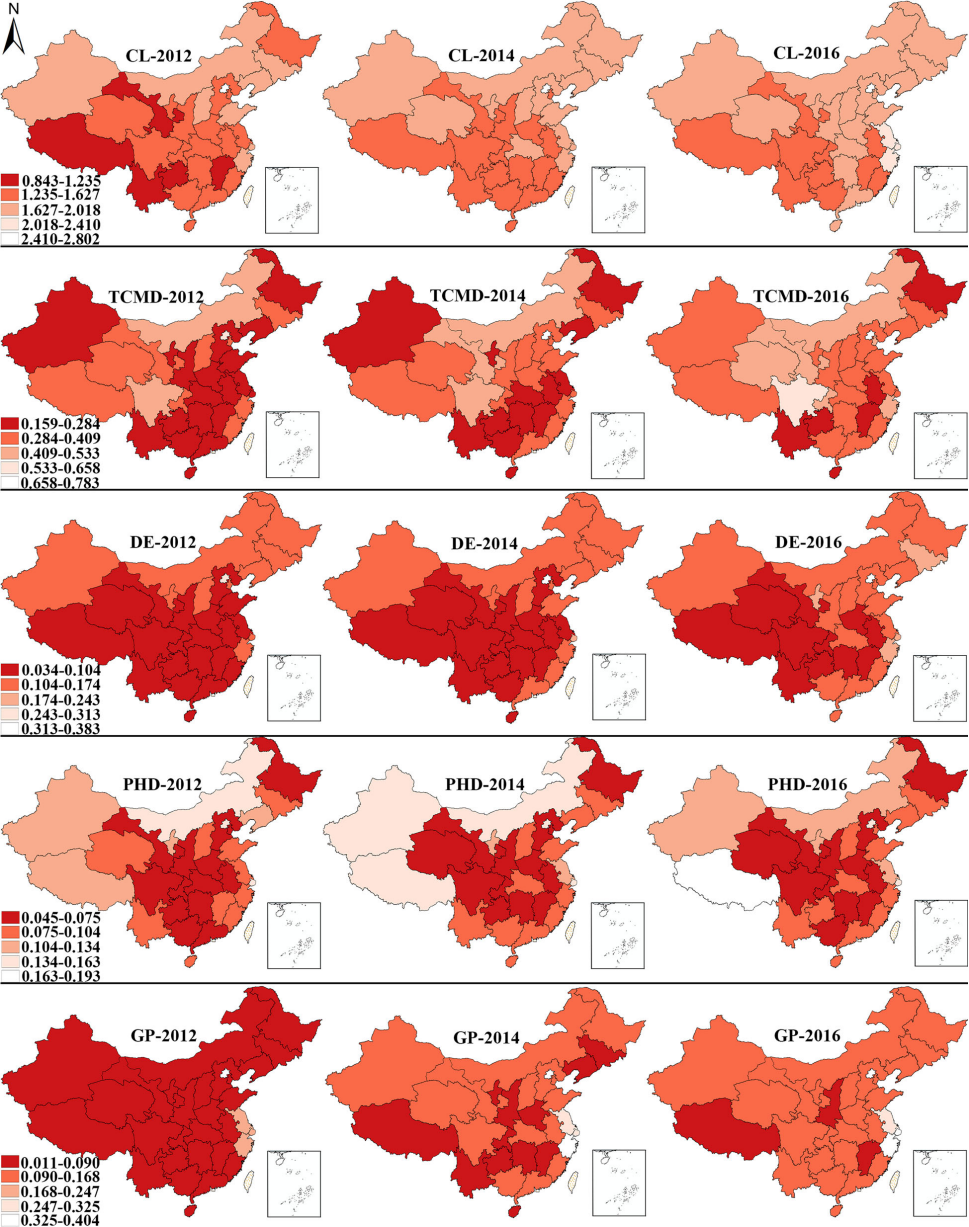
Hierarchical maps of the density of different subtypes of licensed doctors. (Note: CL = clinicians, TCMD = TCM doctors, DE = dentists, PHD = public health doctors, GP = general practitioners)

**Fig. 8:**
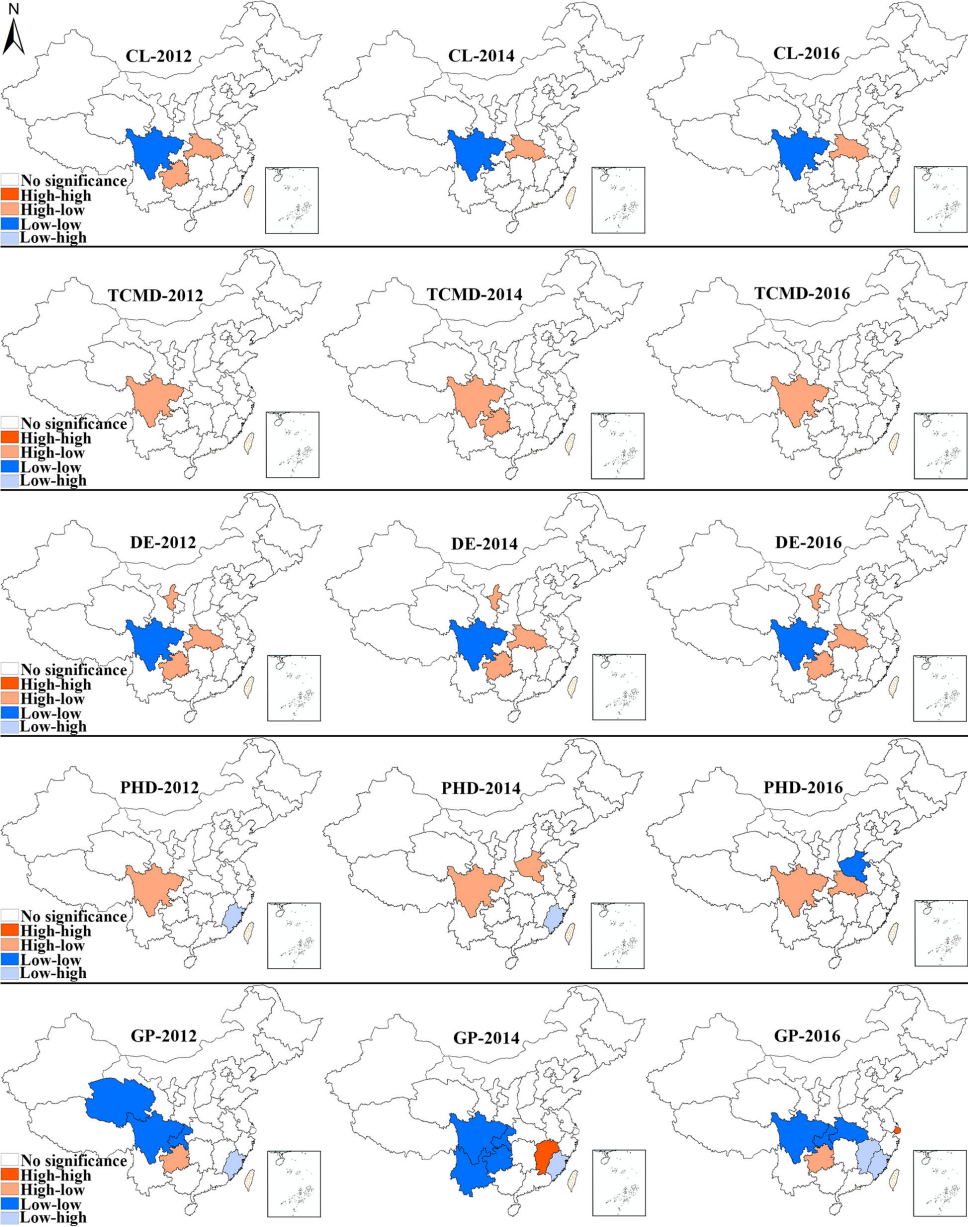
Univariate LISA cluster maps of the density of different subtypes of licensed doctors. (Note: CL = clinicians, TCMD = TCM doctors, DE = dentists, PHD = public health doctors, GP = general practitioners)

Figures 7 and 8 display the hierarchical maps and univariate LISA cluster maps for different subtypes of licensed doctors, respectively. The hierarchical maps in Fig. 7 show the increasing licensed doctor density in almost all the provincial units during 2012–2016. As the changes in color in the hierarchical maps show the change in licensed doctor density quite clearly and vividly, this section will only focus on the interpretations of univariate LISA cluster maps. The clustering pattern of clinicians displayed strong consistency during 2012–2016. Sichuan exhibited low-low cluster feature during 2012–2016. Similarly, the high-low cluster feature in Hubei achieved statistical significance across the whole period. However, Guizhou only exhibited a high-low cluster feature in 2012. The clustering pattern of TCM doctors was much simpler, only the high-low cluster type was detected. Sichuan had been displaying high-low cluster during the whole research period, while the high-low cluster feature in Guizhou was only significant in 2014. The clustering pattern of dentists remained unchanged during the research period. Sichuan had been showing low-low cluster feature at all the three time points, while the high-low cluster type could always be found in three provincial units (Ningxia, Hubei, Guizhou). As for public health doctors, Sichuan was the only provincial unit which was included in the high-low cluster area and significant all the time. Henan and Hubei displayed high-low cluster feature only in 2014 and 2016, respectively. In addition, the low-low type was only detected in Henan in 2016, and the low-high cluster feature was only significant in Fujian at the first two time points. The last but not least, the density of general practitioners in Sichuan and Chongqing had been displaying low-low cluster feature since 2012, while that in Qinghai, Yunnan, Guizhou and Hubei were only significant at certain time points. The only significant high-low cluster area could be found in Jiangxi in 2014. Fujian was identified with a low-low cluster feature at three time points, while Jiangxi turned from high-low cluster feature to low-high during 2014–2016. In the year of 2016, the high-high type and high-low were found in Shanghai and Guizhou, respectively.

## Discussion

Through analyses of a 5-year (2012–2016) panel dataset, this study visualized and compared the temporal trends, spatial variations in temporal trends, and changing spatial patterns of different subtypes of licensed doctors in China. Overall, China has experienced a steady and rapid increase in both licensed doctor quantity and density. However, licensed doctor misdistribution reduced the equity of the health systems in China throughout this period. Thus, scarcity is no longer a problem; uneven distribution is now at the center of discussions.

The present study has shown that the regional differences in the densities of each subtype of licensed doctors remain relatively high, indicating some provincial units are lagging behind. Compared to other regions, the western geographical units are more likely to suffer from licensed doctor shortage [35], which may result in lower and accessibility and efficiency of health services in these units. For example, Sichuan province, which is located in the southeast border area, was low-low cluster overlap area for various subtypes of licensed doctors, indicating a relative lack of licensed doctors in them and adjacent units.

We have reasons to believe that the problem of health manpower misdistribution was primarily due to China’s vast territory. There are 32 administrative units in mainland China, which consist of 21 provinces, 4 municipalities, 5 autonomous regions, and 2 special administrative regions at the province level. They differ in a number of ways. First, in terms of economic strength, the difference in the gross domestic product (GDP) per capita between the richest and the most underdeveloped provinces can be as much as a factor of four, with the eastern region being relatively high in GDP per capita. Second, as for population, the density decreases gradually from the eastern region to the western region, with most people living in the eastern and central regions. Third, differences also exist in geographical features among these administrative units, as the western region has a much vaster territory. Besides, the differences in health expenditure, medical needs and urbanization level may help explain the imbalances in health manpower distribution. In previous studies, Zhu et al. [36] theoretically modeled and empirically measured various determinants of licensed doctor distribution from both the supply and demand sides in China and found that health service demand, government and social health expenditure was, as expected, found to forcefully drive the licensed doctor distribution across the nation. Li et al. [37] found that economic status and urbanization level contributed greatly to the inequalities of licensed doctor distribution in China.

We can draw some take-away lessons from the above-mentioned spatio-temporal dynamics and spatial clusters of the licensed doctor distribution in China.

**First, the units displaying low-low and low-high feature should be given priority in corresponding policy formulation.** Compared with the spread of infectious diseases and air pollutants [38, 39], licensed doctor distribution, affected by multiple geographical, economic, and social factors, shows distinctive spatial clustering features in different areas rather than a single pattern across the whole country. As far as policy planning is concerned, the government should pay extra attention to unit which displayed low-low and low-high cluster feature. The low-low cluster type indicates that the licensed doctor shortage has gone beyond the provincial level and exhibits a trend of regionalization, while the low-high cluster type means that the central unit is at a disadvantage when compared to its neighbors. For the units included in low-low, low-high cluster areas and their neighbors, cooperation, not confrontation, should be strengthened, such as collaborative policy design for the education of medical students and measures to attract licensed doctors [40].

**Second, the pursuit of total quantity balance may obscure the imbalances of certain subtypes of licensed doctors.** Different subtypes of licensed doctors displayed relatively different spatio-temporal dynamics, which may be attributed to their relatively different cultivation channels and job requirements. It also suggests that licensed doctor allocation policies should probably vary across subtypes of licensed doctors. As indicated by the differentiated spatial clustering patterns of different subtypes of licensed doctors, the balanced distribution of total licensed doctors tends to obscure the imbalances of its subtypes. For instance, Sichuan displayed high-low and low-low cluster feature when it comes to different subtypes of licensed doctors, indicating its differentiated position in the distribution of different subtypes of licensed doctors. That is, if we only pursue the balance of total licensed doctor quantity, we may ignore imbalances in its subtypes. However, the current *Long-Term Medical Personnel Development Plan of China (2011–2020)* only sets quantitative goals for total licensed doctors. This practice may cover over imbalances in certain subtypes of licensed doctors.

**Third, the characteristic differences across subtypes of licensed doctors reveal another dimension that is often ignored—skill mix imbalances.** Effective and efficient health service delivery requires not only a sufficient quantity of licensed doctors but also proper teamwork in which each member contributes different skills and performs different functions. The key issue sometimes may not be the quantity but the variety and composition of mixed skilled personnel. In effect, the differences among the spatial clustering patterns of different subtypes of licensed doctors result from the different proportions and variegated composition of licensed doctors in each provincial unit. In both high- and low-income countries and in different medical institutions, there are numerous examples of the mismatch between the capability of the workforce and their current positions [41]. For instance, skilled nurses are doing technologists’ tasks because of a lack of technologists. Practicing medical personnel are burdened with management duties regardless of having no expertise in these areas. This may all lead to low efficiency and excessive workload for certain kinds of health manpower [42]. There is no doubt that an appropriate and sustainable skill mix contributes to more efficient health services delivery in China.

Based on discussions above, this study can help the China government map out more effective strategies in managing the distribution of licensed doctors:

In the short term, the government should make subtype-specific policies for allocating licensed doctors, which gives priority to the identified up-to-date units which displayed low-low and low-high cluster feature. This includes but is not limited to preferential policies to channel the most-needed manpower subtypes to the most-in-need provincial units. More specifically, the identified low-low and low-high clusters should be precisely the priority areas for existing licensed doctor allocation programs in China. For example, the ongoing “Rural-Oriented Medical Education Scheme” (RMES) has been educating licensed doctor for disadvantaged provincial units. The health sector provides financial support for medical students with a rural background, who will in return work in primary-level medical and health care institutions in their hometowns for a couple of years after graduation. This has proven to be an effective strategy to promote the balanced distribution of licensed doctors, as it is much more difficult to urge the licensed doctors elsewhere to migrate to the remote provinces or attract urban licensed doctors to the countryside [43, 44]. The identified cluster areas will further promote RMES to channel the most-in-needed doctor subtype to the most-in-need areas. Besides, it has been a tradition in China to pair some eastern provinces with sufficient skilled licensed doctor stock with disadvantaged western units. The detection of the spatial clusters will make such pairings more accurate by pairing up the provincial units that are most in need and those units which have a comparatively abundant stock of licensed doctors. For the remote regions whose problems cannot be solved by the previous long-term policies, some expediency can be afforded with the assistance of emerging technologies. For example, telemedicine is one possible solution to support the remote or border provinces that have a high demand for some kinds of licensed doctors [45].

In the long run, the results demonstrate the significance and necessity of generating more collaborative efforts involving multiple government sectors (health, financial, etc.) to mobilize domestic resources to combat the uneven distribution of licensed doctors. The Long-Term Medical Personnel Development Plan of China (2011–2020) is currently the only working health manpower plan. Planning the health manpower development every decade has failed to capture the quick changes of health manpower distribution in China and renders the goals of the plan inoperative in the later stages of the planning period. Moreover, it only sets quantitative goals for total health technicians,^1^ total licensed doctors, and total registered nurses, which may obscure imbalances in certain subtypes of health manpower, such as the subtypes of licensed doctors, pharmacists, and technologists. Therefore, a more detailed medical personnel development plan for China that includes each subtype of urban and rural health manpower should be made based on the current status of the health manpower distribution in China. It should set a clear goal for health manpower allocation; be renewable over time; and specify the duties of all the respective government sectors.

This study has some limitations to be acknowledged. Due to data accessibility, this study only focused on the quantity but ignored the quality of licensed doctors [8]. Besides, the underlying causes of licensed doctor misdistribution is not explored in this study. More research is encouraged to delve into this important area and contribute to the better living of mankind, in China as well as in other nations. Future studies are also suggested to center on smaller geographical scales, like the city or county level, and take the quality of licensed doctors into consideration.

## Conclusions

Overall, this study provides cogent evidence for the spatial clustering of different subtypes of licensed doctors in China. First, spatial disparities and spatial clusters exist in the distribution of licensed doctors in China, indicating that some provincial units have been left behind. Second, spatial distribution characteristics vary across different subtypes of licensed doctors.

Just as stated in Healthy China 2030, China would have no national health if without rational distribution of health manpower [46]. This is applied to not only China but also other nations. To maintain the well-being of all citizens regardless of where they live, it requires the equitable distribution of properly trained healthcare workers. Has China reached this goal yet? Our research shows remarkable achievements as well as areas of improvement. In order to achieve a balanced distribution of licensed doctors, the government is suggested to introduce a series of measures, such as deliberative policy design and effective human resource management initiatives to educate, recruit, and retain licensed doctors and prevent a brain drain of licensed doctors from disadvantaged units.

## Supplementary information

**Additional file 1: Figure S1.** The Chinese administrative divisions and their names.

**Additional file 2: Table S1.** The number of different subtypes of licensed doctors in each provincial unit during 2012**–**2016.

**Additional file 3: Table S2.** The number of population in each provincial unit during 2012**–**2016.

## Data Availability

All data generated or analyzed during this study are included in this published article and its supplementary information files

## Abbreviations

CL: Clinicians
DE: Dentists
GDP: Gross domestic product
GP: General practitioners
GP: General practitioners
HH: High-high
HL: High-low
LH: Low-high
LISA: Local indicators of spatial association
LL: Low-low
MDGs: Millennium development goals
PHD: Public health doctors
RMES: Rural-oriented medical education scheme
SDGs: Sustainable development goals
TCM: Traditional chinese medicine
TCMD: Traditional chinese medicine doctors
WHO: World health organization

## Footnotes

1 Health technicians make up the most important component of health manpower and account for three-fourths of the total number of health workers. They comprise licensed doctors, registered nurses, pharmacists, technologists, and interns.

## References

[1] United Nations (2015). Transforming our world: the 2030 agenda for sustainable development.

[2] WHO. Health in 2015: from MDGs to SDGs. Geneva: WHO; 2015.

[3] Doull L, Campbell F (2008). Human resources for health in fragile states. Lancet..

[4] Chen L, Evans T, Anand S, Ivey Boufford J, Brown H, Chowdhury M (2004). Human resources for health: overcoming the crisis. Lancet.

[5] WHO. Reassessing the relationship between human resources for health, intervention coverage and health outcomes. Geneva: WHO; 2006.

[6] WHO. A Universal Truth: No Health Without a Workforce. Geneva: WHO; 2013.

[7] Shamian J, Murphy GT, Rose AE, Jeffs L (2015). Human resources for health: a new narrative. Lancet.

[8] Anand S, Fan VY, Zhang J, Zhang L, Ke Y, Dong Z (2008). China’s human resources for health: quantity, quality, and distribution. Lancet.

[9] National Health and Family Planning Commission of the PRC (2018). China health and family planning statistical yearbook 2018.

[10] National Health and Family Planning Commission of the PRC. Long-term medical personnel development plan (2011-2020). Beijing; 2011.

[11] Asante AD, Zwi AB, Ho MT (2006). Equity in resource allocation for health: a comparative study of the Ashanti and northern regions of Ghana. Health Policy (New York).

[12] Tokuhata GK, Newman P, Digon E, Mann LA, HArtman T, Ramaswamy K (1975). Health manpower distribution in Pennsylvania. Am J Public Health.

[13] Ahmed SM, Hossain MA, RajaChowdhury AM, Bhuiya AU (2011). The health workforce crisis in Bangladesh: shortage, inappropriate skill-mix and inequitable distribution. Hum Resour Health.

[14] Gupta N, Zurn P, Diallo K, Poz MRD (2003). Uses of population census data for monitoring geographical imbalance in the health workforce: snapshots from three developing countries. Int J Equity Health.

[15] Hazarika I (2013). Health workforce in India: assessment of availability, production and distribution. WHO South-East Asia J Public Heal.

[16] Theodorakis PN, Mantzavinis GD, Rrumbullaku L, Lionis C, Trell E (2006). Measuring health inequalities in Albania: a focus on the distribution of general practitioners. Hum Resour Health.

[17] Honarmand R, Mozhdehifard M, Kavosi Z (2017). Geographic distribution indices of general practitioners, midwives, pediatricians, and gynecologists in the public sector of Iran. Electron Physician.

[18] Wiseman V, Lagarde M, Batura N, Lin S, Irava W, Roberts G (2017). Measuring inequalities in the distribution of the Fiji Health workforce. Int J Equity Health.

[19] Chen R, Zhao Y, Du J, Wu T, Huang Y, Guo A (2014). Health workforce equity in urban community health service of China. PLoS One.

[20] Liu W, Liu Y, Twum P, Li S (2016). National equity of health resource allocation in China: data from 2009 to 2013. Int J Equity Health.

[21] WHO. Establishing and monitoring benchmarks for human resources for health: the workforce density approach. Genova, Switzerland; 2008. http://www.who.int/hrh/statistics/spotlight/en/index.html. Accessed 21 Feb 2020.

[22] Altman I (1961). Changes in physician-population ratios among the states. Public Health Rep.

[23] Bigbee JL, DeMeyer J (2007). The relationship between nurse to population ratio and population density: a pilot study in rural/frontier state. Online J Rural Nurs Heal Care.

[24] Dal Poz MR, Gupta N, Quain E, Soucat ALB. Handbook on monitoring and evaluation of human resources for Health: with special applications for low- and middle-income countries. Geneva: WHO; 2009.

[25] Shetty A, Shetty S (2014). The correlation of physician to population ratio and life expectancy in Asian countries. J Med Sci Clin Res.

[26] Huang L, Li X-X, Abe EM, Xu L, Ruan Y, Cao C-L (2017). Spatial-temporal analysis of pulmonary tuberculosis in the northeast of the Yunnan province. People’s Repub China Infect Dis Poverty.

[27] Zhu B, Liu J, Fu Y, Zhang B, Mao Y (2018). Spatio-temporal epidemiology of viral hepatitis in China (2003–2015): implications for prevention and control policies. Int J Environ Res Public Health.

[28] Fischer MM, Griffith DA (2008). Modeling spatial autocorrelation in spatial interaction data: An application to patent citation data in the european union. J Reg Sci.

[29] Anselin L (1995). Local indicators of spatial association—Lisa. Geogr Anal.

[30] Hajizadeh M, Campbell MK, Sarma S (2016). A spatial econometric analysis of adult obesity: evidence from Canada. Appl Spat Anal Policy.

[31] Long R, Shao T, Chen H (2016). Spatial econometric analysis of China’s province-level industrial carbon productivity and its influencing factors. Appl Energy.

[32] Cheng Y, Wang Z, Ye X, Wei Y (2014). Spatiotemporal dynamics of carbon intensity from energy consumption in China. J Geogr Sci.

[33] Li Q, Song J, Wang E, Hu H, Zhang J, Wang Y (2014). Economic growth and pollutant emissions in China: a spatial econometric analysis. Stoch Environ Res Risk Assess.

[34] Anselin L, Syabri I, Kho Y (2006). Geoda: an introduction to spatial data analysis. Geogr Anal.

[35] Zhu B, Hsieh C-W, Zhang Y (2018). Incorporating spatial statistics into examining equity in Health workforce distribution: an empirical analysis in the Chinese context. Int J Environ Res Public Health.

[36] Zhu B, Hsieh C, Mao Y. Addressing the licensed doctor Maldistribution in China : a demand-and-supply perspective. Int J Environ Res Public Health. 2019. 10.3390/ijerph16101753 PMC6571941 31108920

[37] Li D, Zhou Z, Si Y, Xu Y, Shen C, Wang Y (2018). Unequal distribution of health human resource in mainland China: what are the determinants from a comprehensive perspective?. Int J Equity Health.

[38] Zhu B, Fu Y, Liu J, Mao Y (2017). Notifiable sexually transmitted infections in China: epidemiologic trends and spatial changing patterns. Sustainability.

[39] Hao Y, Liu YM (2016). The influential factors of urban PM2.5 concentrations in China: a spatial econometric analysis. J Clean Prod.

[40] Shadmi E, Wong WCW, Kinder K, Heath I, Kidd M (2014). Primary care priorities in addressing health equity: summary of the WONCA 2013 health equity workshop. Int J Equity Health.

[41] WHO. World Health Report 2006: working togehther for health. Geneva: WHO; 2006.

[42] Fulton BD, Scheffler RM, Sparkes SP, Auh EY, Vujicic M, Soucat A (2011). Health workforce skill mix and task shifting in low income countries: a review of recent evidence. Hum Resour Health.

[43] Somers GT, Strasser R, Jolly B (2007). What does it take? The influence of rural upbringing and sense of rural background on medical students’ intention to work in a rural environment. Rural Remote Health.

[44] King KR, Purcell RA, Quinn SJ, Schoo AM, Walters LK (2016). Supports for medical students during rural clinical placements: factors associated with intention to practise in rural locations. Rural Remote Health.

[45] Cai H, Wang H, Guo T, Bao G (2016). Application of telemedicine in Gansu Province of China. PLoS One.

[46] Wang W, Zakus D (2016). Healthy China 2030: “Without national health, there will be no comprehensive well-being.”. Fam Med Commun Heal.

