# Supplementary figures and images for: Spatio-temporal variations of licensed doctor distribution in China: measuring and mapping disparities
Source: BMC Health Serv Res. 2020 Mar 2;20:159. doi: 10.1186/s12913-020-4992-2 (PMC7053041; doi:10.1186/s12913-020-4992-2)

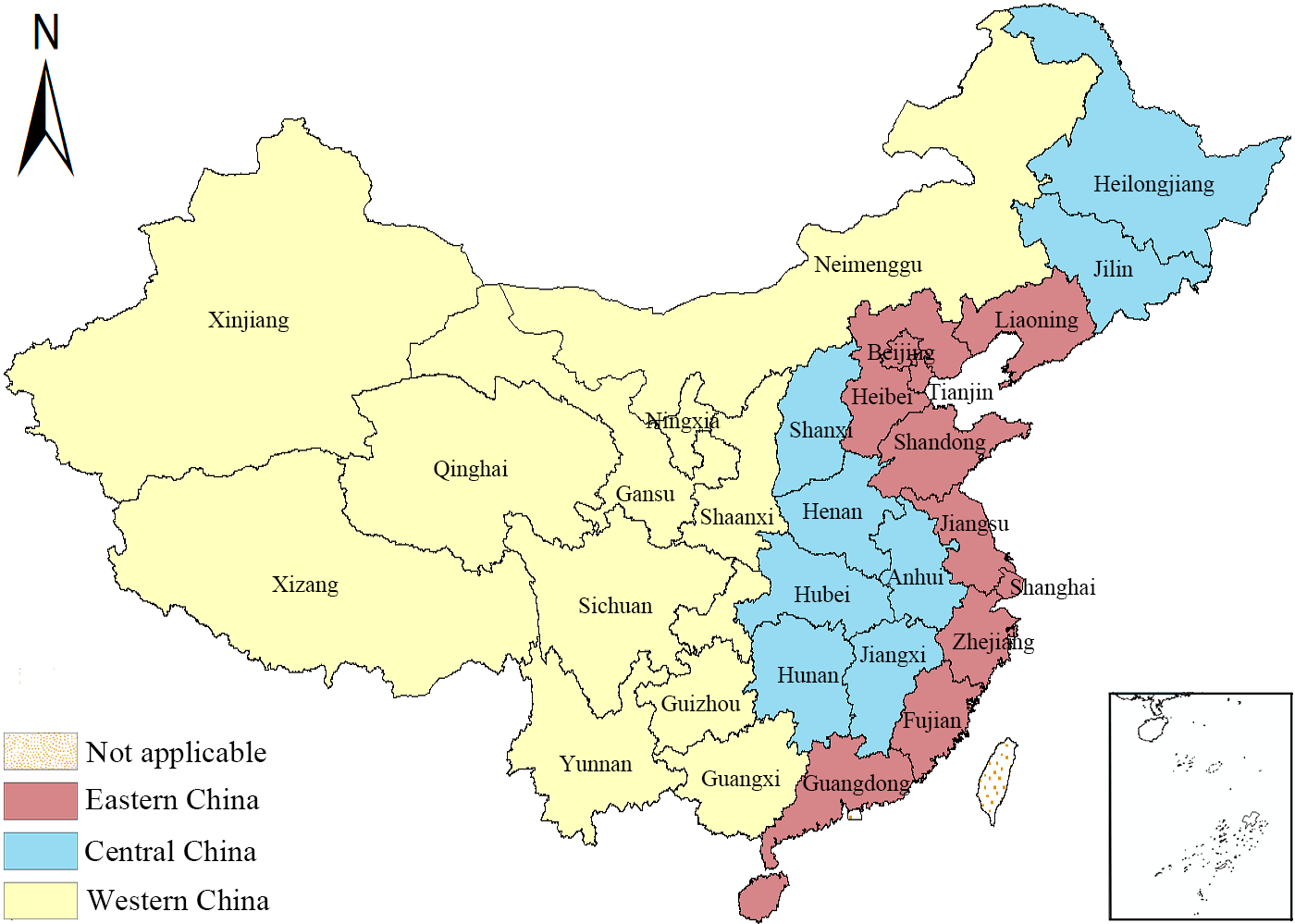

Supplement: Supplementary file 1 — Additional file 1: Figure S1. The Chinese administrative divisions and their names. [file 12913_2020_4992_MOESM1_ESM.docx]
